# Induction of apoptosis by a peptide from *Porphyra yezoensis*: Regulation of the insulin-like growth factor I receptor signaling pathway in MCF-7 cells

**DOI:** 10.3892/ijo.2014.2509

**Published:** 2014-06-20

**Authors:** SU-JIN PARK, JINA RYU, IN-HYE KIM, YOUN-HEE CHOI, TAEK-JEONG NAM

**Affiliations:** 1 Department of Food and Science, Pukyong National University, Busan 608-737, Republic of Korea; 2 Institute of Fisheries Science, Pukyong National University, Busan 619-911, Republic of Korea

**Keywords:** *Porphyra yezoensis*, peptide, anticancer, apoptosis, cell cycle

## Abstract

This study examined how PPY, a peptide from *Porphyra yezoensis*, regulates multiple cell growth-related signaling pathways in MCF-7 cells. This study determined that PPY induces cell cycle arrest and inhibits the IGF-IR signaling pathway. Cell proliferation studies revealed that PPY induced cell death in a dose-dependent manner. Expression levels of IGF-IR were decreased in MCF-7 cells by PPY in a dose-dependent manner. These results indicate that inhibition of the IGF-IR pathway is also involved in PPY induced proliferation of MCF-7 cells. In addition, these data demonstrated that PPY induces cell cycle arrest and activates apoptosis.

## Introduction

Different species of seaweed have received a great deal of attention from researchers in recent years. Seaweeds contain high amounts of proteins, vitamins and minerals, and several polysaccharides found in seaweed have been shown to exhibit diverse biological activities. In particular, the antitumor and antibacterial activities of seaweed have been widely studied; furthermore, effects on the immune system have been demonstrated (1,2). *Porphyra yezoensis* is an intertidal marine red algae that has received increasing attention as a model organism, owing to its important role in biological research (3). *Porphyra yezoensis* is one of the most important edible seaweeds, and accordingly, is one of the most valuable marine crops in the world; it is cultivated widely in Asia, especially in Japan, China and Korea (4). Although several studies have examined the polysaccharides found in the extracts of *Porphyra yezoensis*, the effects of particular proteins have not been reported. The peptide PPY from *Porphyra yezoensis* is known to play a role in antitumor cell signaling, but the mechanism behind this activity is not well understood. Insulin-like growth factor I (IGF-I) and its cognate receptor, insulin-like growth factor I receptor (IGF-IR), play important roles in normal cell function and tumorigenesis, via their mediation of cell growth, differentiation and survival (5); numerous studies have shown that overexpression of IGF-IR and related proteins results in cancer cell proliferation and survival (6–8). The role of IGF-I signaling in tumor growth has been demonstrated *in vivo* using nucleic-acid based strategies.

Apoptosis, the process of active programmed cell death, occurs under many important physiological conditions, and it is a critical part of normal development and differentiation in a wide variety of tissues. This form of cell death has been extensively studied in cancer research as a potential mechanism by which the body eliminates precancerous and/or cancerous cells (9). Apoptosis is characterized by several unique features, including cell shrinkage, chromatin condensation, DNA fragmentation, the expression of phosphatidylserine on the cell surface and membrane blebbing (10,11).

Cyclins are key cell cycle control molecules with specific and periodic expression associated with cell cycle progression (12). Other cell cycle control proteins include cyclin-dependent kinase (cdk) inhibitors, such as p21 and p27, which tightly regulate the activities of cyclin/cdk enzyme complexes (13). Mitogen-activated protein kinases (MAPKs), another important class of proteins, are activated in response to a wide variety of extracellular stimuli and mediate signal transduction cascades that play important roles in cell proliferation, differentiation, cell cycle control and apoptosis (14).

It has been shown that PPY has antitumor effects, and the important role of IGF-I in mediating numerous cell survival pathways is also well established. This study aimed to determine whether PPY induces apoptosis via IGF-IR signaling.

## Materials and methods

### Preparation of peptide

The peptide PPY, found in *Porphyra yezoensis*, was synthesized by the Peptron (Daejeon, Korea). Purification of PPY was performed using a Shimadzu Prominence HPLC apparatus and controlled using the software package Class-VP, 6.14 (Kyoto, Japan). A C18 column (Shiesido Capcell Pak) in 0.1% TFA/water and a gradient of 10–70% acetonitrile in 0.1% TFA, with a flow rate of 1 nm/min and UV detection at 220 nm was used.

### Cell culture

MCF-7 human breast cancer cells were obtained from the Korean Cell Line Bank (KCLB) and grown in RPMI-1640 medium supplemented with 10% fetal bovine serum, 100 μg/ml penicillin and 100 ng/ml streptomycin at 37°C in a humidified atmosphere with 5% CO_2_.

### Cell proliferation assay

Cell proliferation was estimated using a CellTiter 96 aqueous non-radioactive cell proliferation assay (Promega, Madison, WI, USA), which is based on the cleavage of 3-(4,5-dimethylthiazol-2-yl)-5-(3-carboxymethoxy-phenyl)-2H-tetrazolium (MTS) into a formazan product that is soluble in cell culture medium. Cells were seeded onto 96-well plates at 2×10^4^ cells per well in 100 μl medium and allowed to attach for 24 h. The cell monolayer was washed with phosphate-buffered saline (PBS) to remove unattached cells. The attached cells were maintained in serum-free medium (SFM) for 12 h and then washed with PBS. Cells were then incubated with fresh SFM containing various concentrations (0–500 ng/ml) of peptide for 24 h. Subsequently, the cells were incubated with 10 μg/ml MTS solution for 30 min, and the absorbance of each well was measured at 490 nm using a SpectraMAX 340-pc multi-plate reader (Molecular Devices, Sunnyvale, CA, USA).

### DAPI staining assay

Cell were washed with PBS and fixed with 3.7% paraformaldehyde in PBS for 10 min at room temperature. Fixed cells were washed with PBS and stained with 2.5 μg/ml 4,6-diamidio-2-phenylindole (DAPI) solution for 10 min at room temperature. The cells were washed twice with PBS and analyzed using a fluorescent microscope.

### Western blot analysis

Proteins (50 μg/ml) from cell lysate were separated using 7.5–15% SDS-PAGE and transferred to a polyvinylidene fluoride (PVDF) membrane (Millipore, Billerica, MA, USA). The membranes were blocked with 1% bovine serum albumin (BSA) in TBS-T (10 mM Tris-HCl, 150 mM NaCl, pH 7.5, 0.1% Tween-20) and then incubated overnight with the indicated primary antibodies (diluted 1:1,000) in TBS-T containing 1% BSA with gentle shaking at 4°C. The secondary antibody was peroxidase-conjugated goat anti-mouse or anti-rabbit (diluted 1:10,000). Signals were detected using an ECL western blotting kit (Amersham, Piscataway, NJ, USA).

### Apoptosis assay

The Annexin V and Dead Cell Assay was performed utilizing the Muse™ Cell Analyzer from Millipore following the manufacturer’s instructions. Briefly, after the indicated treatments, the cells were incubated with Annexin V and Dead Cell Reagent (7-aminoactinomycin D; 7-AAD), and the dead/late apoptotic, early apoptotic and live cells were counted.

## Results

### Synthesized peptide from Porphyra yezoensis

**Figure 1:**
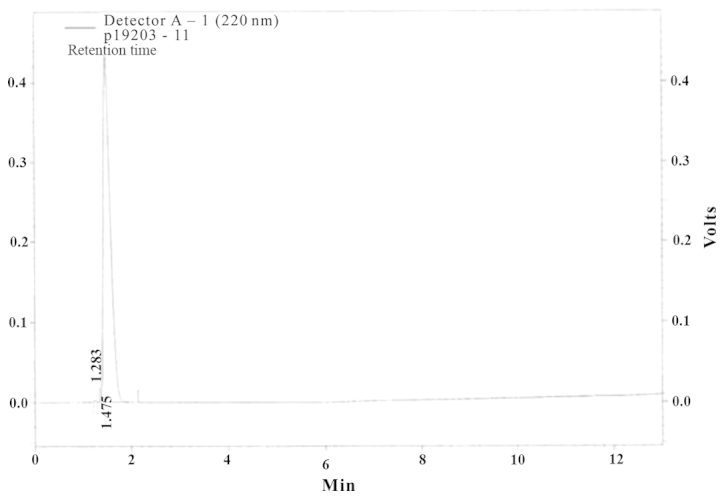
Separation of peptide *Porphyra yezoensis* (PPY). Separation of peptide PPY by HPLC (capcell pak C18 column).

Purified peptides were analysis as KKAAE, and the molecular mass was determined to be 546 Da (Fig. 1).

### Inhibitory effect of PPY on proliferation of MCF-7 cells

**Figure 2:**
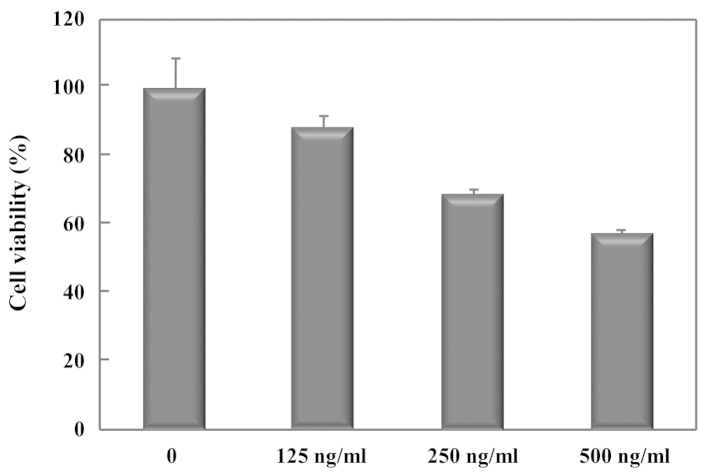
Effect of PPY on proliferation of MCF-7 cells. MCF-7 cells were treated with various concentrations of PPY for 24 h and cytotoxicity was evaluated using the MTS assay.

**Figure 3:**
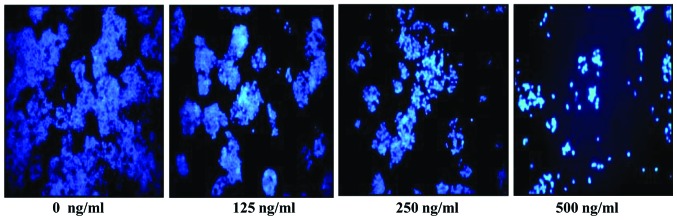
Effect of PPY on cell morphology of MCF-7 cells. PPY caused morphological changes in MCF-7 cancer cells, as assessed using DAPI staining. After incubation with PPY (0–500 ng/ml) for 24 h, cells were observed under an optical microscope. Photographs were taken at a magnification of ×200.

PPY inhibited proliferation of MCF-7 breast cancer cells, as determined by the MTS assay. This assay revealed that PPY induced growth inhibition occurred in a dose-dependent manner (Fig. 2), and treatment with the highest concentration of peptide (500 ng/ml) for 24 h resulted in 60% inhibition of cell growth. In addition to growth inhibition, PPY treatment of MCF-7 cells decreased the relative cell numbers, which was also concentration-dependent manner. This decrease is attributable to the induction of apoptotic cell death by PPY, as determined by a DAPI assay (Fig. 3). These conclusions were further supported through cell morphology observations, which indicated that cells treated with PPY revealed to decrease in number compared with untreated cells. DAPI staining showed that PPY inhibited the proliferation of MCF-7 cells in a time-dependent manner. The DAPI assay also confirmed that PPY treatment induced cell death. Taken together, these results demonstrate that PPY induces both growth inhibition and apoptosis in MCF-7 cells.

### PPY induced phosphorylation of the PI3K-Akt pathway

**Figure 4:**
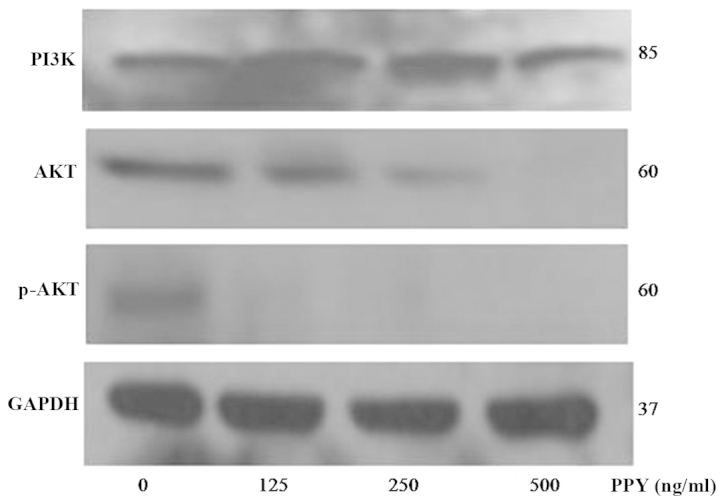
Effect of PPY on the expression of levels of the PI3K/Akt pathway. Cells were treated with various concentrations of PPY (0–500 ng/ml) for 24 h. PI3K, AKT and p-AKT protein levels are shown.

The phosphatidylinositol 3-kinases (PI3K)/Akt pathway is mainly associated with cell growth and is a critically important regulator of cell differentiation and proliferation. This prompted us to examine the potential involvement of this pathway in PPY induced inhibition of MCF-7 cell proliferation. This study examined whether PPY influenced the activation of p85, a subunit of PI3K. MCF-7 cells treated with PPY had a decreased level of Akt phosphorylation/activation, compared with untreated cells. Moreover, PPY treatment decreased the activation of p85 (Fig. 4). These results suggest that the inhibition of the PI3K/Akt pathway is at least part of the mechanism by which PPY inhibits MCF-7 cell proliferation.

### PPY affects the expression of IGF-IR binding proteins in MCF-7 cells

**Figure 5:**
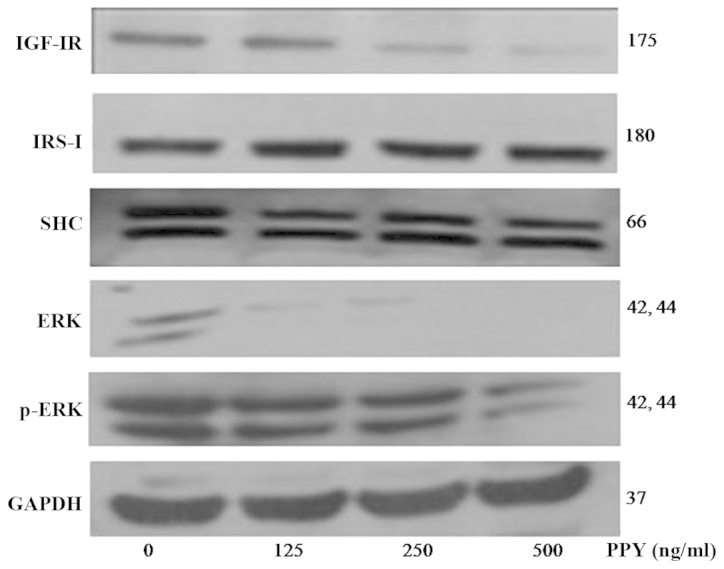
Effect of PPY on the expression of levels of the IGF-I signaling pathway. Cells were treated with various concentrations of PPY (0–500 ng/ml) for 24 h. IGF-IR, IRS-I, SHC, ERK and p-ERK protein levels are shown.

To further investigate the mechanism of PPY induced growth inhibition, additional components of the IGF signaling pathway were examined. The signaling activity of IGF-IR is a crucial regulator of apoptosis and cell proliferation and IGF-IR activation results in auto-phosphorylation (8). Therefore, the effect of PPY on IGF-IR expression was examined. MCF-7 cells were treated with different concentrations of PPY for 24 h; the protein expression of apoptosis-associated proteins is shown in Fig. 4. Expression levels of IGF-IR and IRS-I were decreased by PPY treatment in a dose-dependent manner. Total ERK protein expression decreased in cells treated with 125 ng/ml PPY, and phosphorylation of ERK was inhibited by PPY. Consistent with these findings, treatment with PPY resulted in activation of the intrinsic apoptosis pathway, which can be induced by decreased ERK and phospho-ERK in MCF-7 cells (Fig. 5). Induction of apoptosis is a predominant mechanism by which chemotherapeutic agents exert cytotoxicity (15). The data presented here provide evidence for the apoptotic effect of PPY in MCF-7 cells.

### PPY affects the expression of cell cycle-related proteins in MCF-7 cells

**Figure 6:**
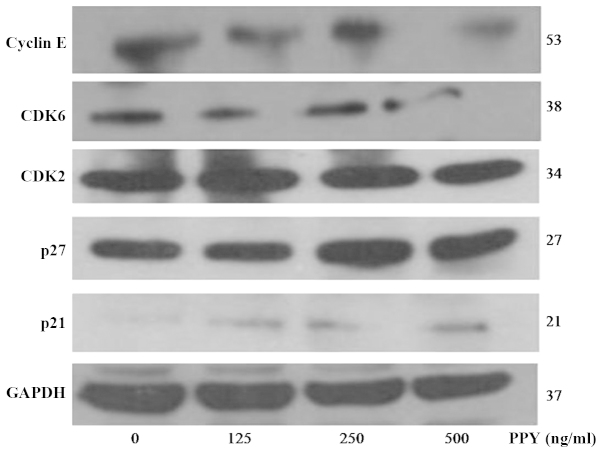
Effect of PPY on the expression of cell cycle proteins. Cells were treated with various concentrations of PPY (0–500 ng/ml) for 24 h. Cyclin E, cdk6, cdk2, p27 and p21 protein levels are shown.

Cell cycle progression is a highly ordered and tightly regulated process that involves multiple checkpoints that monitor extracellular growth signals, cell size and DNA integrity. Deregulation of the cell cycle has been recognized as a hallmark of cancer progression in most malignant tumors. Furthermore, cell cycle deregulation has been shown to induce an aberrant form of mitosis called mitotic catastrophe and it may also be involved in triggering apoptosis (3). The cdk inhibitors p21 and p27 suppress the activity of the pro-proliferative cyclin E/Cdk complex. Cyclin E is highly expressed during the G1 to S phase transition, and Cdks, including Cdk2, Cdk4, Cdk6, are critical regulators during phase transitions. Accordingly, we examined whether PPY treatment affected critical cell cycle regulators. Incubation of MCF-7 cells with PPY resulted in a dose-dependent decrease in the expression levels of the pro-proliferative proteins cyclin E and cdk6, while levels of Cdk2 were largely unchanged. In contrast, expression of p21 and p27 increased in response to PPY treatment dose-dependently (Fig. 6). Given that reduced expression of p27 has been observed in several human cancers, and p27 is associated with the induction of apoptosis in cancer cells (16), our findings suggest that PPY may modulate the sub-G1 arrest via upregulation of p21 and p27 and downregulation of Cdk6 and cyclin E.

### PPY induces apoptosis in MCF-7 cells

**Figure 7:**
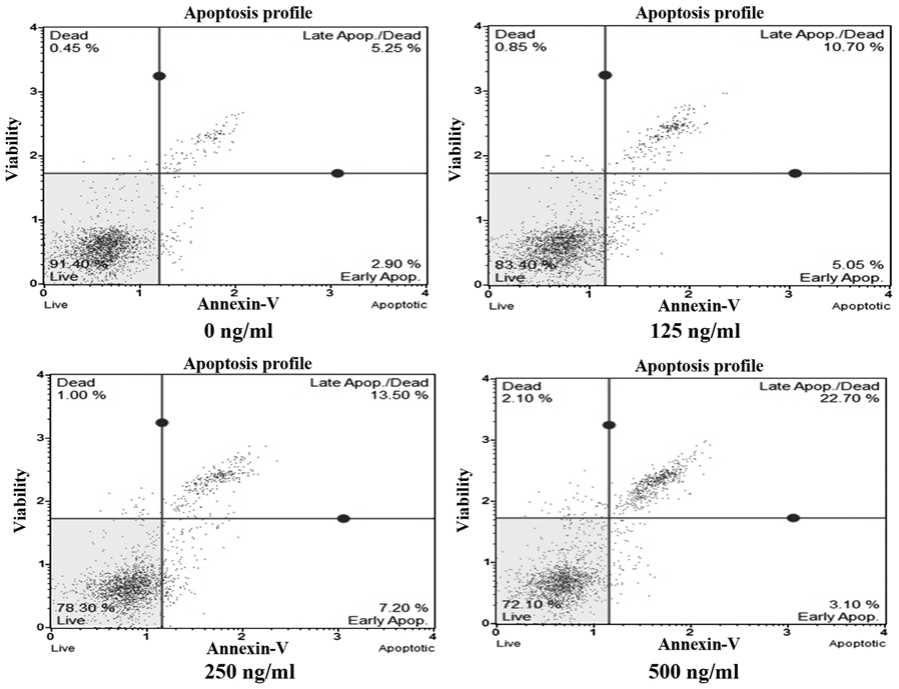
PPY induces apoptosis in MCF-7 cells. Cells cultured in 6-well plates were treated with PPY (0–500 ng/ml) and then collected in medium containing 1% FBS. The percentages of apoptotic and necrotic cells were determined using the Muse Annexin V and Dead Cell kit as described in Materials and methods. Cells in the early stage of apoptosis were Annexin V-positive and 7-AAD-negative, while those in late apoptosis were Annexin V-positive and 7-AAD-positive.

During early apoptosis, the externalization of the phospholipid phosphatidylserine occurs at the cell membrane and can be detected by Annexin V; this reagent can therefore be used to detect cells undergoing apoptosis (17). Using an Annexin V/7-AAD apoptosis assay, PPY treatment was found to induce apoptosis of MCF-7 cells in a dose-dependent manner. A control cell population was comprised of 8.15% apoptotic cells, 0.45% necrotic cells and 91.40% living cells (Fig. 7). Treatment with 125, 250 and 500 ng/ml PPY for 24 h resulted in 15.75, 20.70 and 25.80% apoptotic cells, respectively. This indicates that even at the highest concentration, cell death via apoptosis still occurs, which is desirable from a chemotherapeutic standpoint.

**Figure 8:**
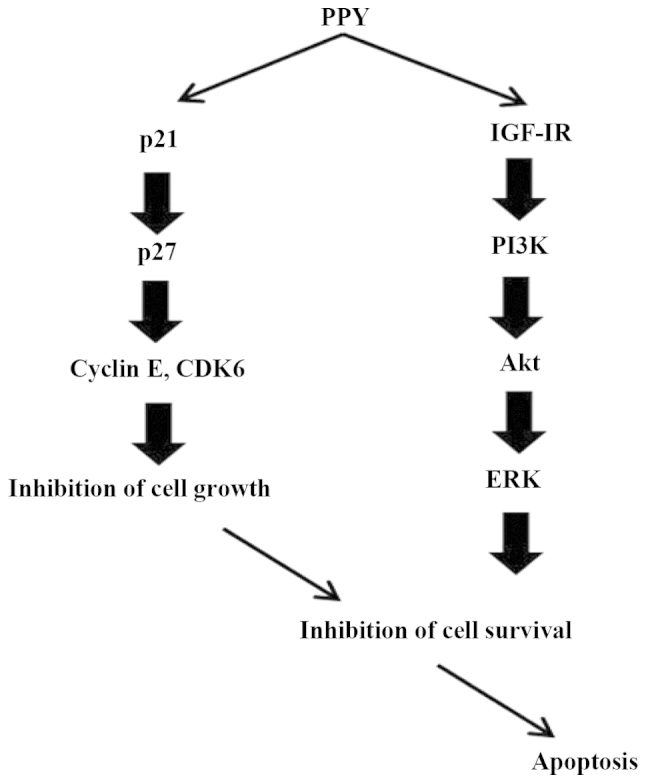
Proposed model of PPY induced apoptosis in MCF-7 cells.

In conclusion, this study investigated the effective PPY on the inhibition of MCF-7 cell proliferation, as well as the possible mechanisms of growth inhibition. This study showed PPY apoptotic cells and identified regulation of the IGF-IR signaling pathway in MCF-7 cells (Fig. 8).

## Discussion

Various seaweed types have high levels of nutrients and other potentially beneficial components that may be useful for the treatment of various diseases. In particular, the antitumor and antibacterial activities of seaweeds have been widely studied (2).

The aim of present study was to determine whether PPY could inhibit the growth of MCF-7 breast cancer cells, as well as the underlying mechanism. Several mechanisms were identified. First, treatment of MCF-7 cells with PPY induced the cells to undergo apoptosis, which was confirmed by examining the nuclear morphology (using a DAPI staining assay). The cells treated with PPY appeared to decrease in number compared with untreated cells. This observation was confirmed using an apoptosis assay that quantitatively demonstrated that PPY induces apoptosis in MCF-7 cells dose-dependently.

In addition to apoptosis, cell population size is also influenced by regulation of proliferation. Stimulation of cell proliferation and division is mediated by multiple signaling pathways induced by tyrosine kinases, including MAPK and PI3K (18). Tyrosine kinase receptors transduce extracellular growth signals to the nucleus using signal transduction pathways, including the Ras/Raf/MAPK and PI3K/Akt pathways (22). PI3K is activated by the growth factor EGF (19), and following its activation, Akt is recruited to the cytoplasmic surface of the cell membrane (20,21). This study demonstrates that, in addition to inducing apoptosis, PPY also inhibits the PI3K/Akt pathway in MCF-7 cells. The PI3K/Akt pathway has been identified as key player in cell survival (23,24). Akt also functions in normal growth, as evidenced by Akt-knockout mice, which show retarded growth (25,26).

The variety of ways in which apoptosis can be induced suggests that wild-type IGF-IR and its ligands may have widespread anti-apoptotic effects via many death signals (27). IGF-IR levels have been found elevated in breast cancer compared with non-malignant tumors or normal epithelium (28). In breast cancer cell lines, IGF-IR is often co-expressed with autocrine IGF-like mitogens that promote cell proliferation (29). The present study indicates that PPY induces apoptosis in MCF-7 cells by downregulating the expression of IGF-IR and IRS-I, which initiates the extrinsic apoptosis pathway and the active forms of SHC were detected. Signaling through IGF-IR stimulates proliferation and promotes angiogenesis and metastasis, and there is now abundant evidence indicating that signaling through the IGF-IR pathway is important for survival of breast cancers as well as cancer cell lines, such as MCF-7 cells (30). Our results show that PPY treatment was effective for growth inhibition and induction of apoptosis in MCF-7 cells.

A model regarding gossypol-induced cell cycle arrest of breast cancer cells has been proposed, which involved the p53, p21, cyclin D1 and Rb cell cycle proteins (31). In response to DNA damage, the cell cycle checkpoints and cell death signals are activated to stop cell growth and to halt the genetically modified cells from multiplying. Damaged cells stop DNA replication at the G1 phase, presumably activating the repair system before the next cell cycle begins (16). Cell cycle progression is a highly ordered and tightly regulated process that involves the sequential activation and inhibition of cyclin-Cdk complexes. There is accumulating evidence that manipulation of the cell cycle may prevent or induce an apoptotic response depending on the cellular context (32). The accumulation of sub-G1 phase cells induced by PPY treatment led us to examine the expression levels of cell cycle regulators. The observed upregulation of Cdk inhibitors and downregulation of cyclin E and Cdk6 reveal a mechanistic explanation for the growth inhibitory effects of this peptide.

In conclusion, our studies investigated the effects of the PPY peptide on the growth of MCF-7 cells, and we have shown here that PPY inhibits MCF-7 cell growth by inducing apoptosis, inhibiting proliferative signaling by antagonizing the IGF-I, and inducing cell cycle arrest by altering expression of key cell cycle modulators.
